# Factors related to changes in severity among adult and older adult patients at an internal medicine department clinic: an embedded mixed-method study

**DOI:** 10.1038/s41598-024-54266-8

**Published:** 2024-02-16

**Authors:** Patcharawan Narongsanoi, Samoraphop Banharak, Ladawan Panpanit, Sutin Chanaboon, Jintana Damkliang

**Affiliations:** 1 Maharat Nakhon Ratchasima Hospital, Nakhon Ratchasima, Thailand; 2 Department of Gerontological Nursing, Faculty of Nursing, Khon Kaen University, Khon Kaen, Thailand; 3 Sirindhorn College of Public Health Khon Kaen, Khon Kaen, Thailand; 4 Department of Adult and Gerontological Nursing, Faculty of Nursing, Prince of Songkla University, Hat Yai, Thailand

**Keywords:** Aging, Emergency medicine, Emergency treatment, First aid, Triage, Diseases, Health care, Medical research, Risk factors, Signs and symptoms

## Abstract

The changes in aging plus the pathology of diseases can influence the changes in severity levels. This study aimed to examine the changes in levels of severity in patients while waiting to see a doctor. The study was conducted at an outpatient clinic in northeastern Thailand with a total of 421 patients who were assessed twice for levels of severity using the Emergency Severity Index. The 38 triage nurses screened patients, and 18 were interviewed when severity level changes were observed. Data were collected April 1–30, 2021. Quantitative data were analyzed by Chi-square test, Fisher's exact test, and logistic regression. Qualitative data were analyzed by content analysis. Most patients were female, between 18 and 59 years old. Most patients did not change their level of severity. However, increasing levels of severity were found in older adults. Factors related to the changes in severity levels were age group, chronic disease, chief complaint, educational level, the duration of travel to the outpatient clinic, type of vehicle, aging process and comorbidity, pathology of diseases, reassessment interval, nurse's experience, bypassing the patient triage process, patient's self-preparation, management of triage nurses, and assignment of direct healthcare staff until the end of the treatment. Increased severity was more frequently found in older adults, so closely monitored during waiting times at a clinic is needed. Setting rescreening as a policy and having sensitive screening guidelines and tools specific to older adults would contribute to early detection and immediate treatment of deteriorating symptoms and illness to help reduce complications and morbidity.

*Trial registration*: https://osf.io/fp3j2.

## Introduction

As people age, the signs and symptoms of illness and responses to treatment are affected by the normal physiological changes of the aging process. These changes and responses in older people become more pronounced during illness and can be differentiated from those of adults^1^. When nurses need to evaluate an older person, there is an acronym that helps to remind them what to consider in the assessment. The acronym RAMPS describes five clinical features associated with aging in older adults who present with an illness: R is for the reduced body reserve in which there is a decline in the body’s energy reserve; A is for the atypical presentation of signs and symptoms for various diseases that are nonspecific to older adults; M is for the multiple pathologies and chronic problems that may be present; P is for polypharmacy or the concurrent use of multiple medications; and S is for the social adversities or unfavorable social changes that may occur as a consequence of aging^2^.

## Background

The changes in aging associated with RAMPS, especially an atypical presentation and reduced body reserve, can influence the nurses’ assessment and determination of a level of illness severity. The atypical presentation in older adults may differ from adults and affect patient screening. For example, pneumonia without fever or acute myocardial infarction without chest pain complicates problem identification in making a diagnosis. Older adults with reduced body reserve become sick more easily, report more severe or worsening symptoms, and experience fluctuations in their symptoms. When older adults are waiting for medical care services, their instability of symptoms can influence nurses’ assessments of severity levels compared to adults in a similar situation^1,3^.

A common issue for health care in outpatient clinics and hospital emergency services is the crowding and congestion of people waiting to be seen. This occurs when the immediate demand for health care services exceeds the supply of medical resources. In these situations, healthcare service capacity is limited. For example, the ratio of patients to populations who visited emergency rooms in 2020 was 421:1,000 in the United States, 412:1,000 in England, 331:1,000 in Australia, and 458:1,000 in Thailand^4^. In the same year, patients in Thailand visited outpatient departments 164 million times, for an average of 3.45 times/person/year, or approximately 679 per 1,000 population^5^. These large numbers resulted in congestion in service areas and a delay in receiving medical treatment. Outpatient clinics have particularly longer wait times than emergency departments, and this may result in a change in symptoms in older adults and an increase in illness severity and mortality outcomes^6,7^.

Triage nurses need to be able to distinguish the differences between adults and older adults during classifying their severity of illness because it is easier for older adults to experience changes in levels of severity while waiting for treatment^7,8^. Lack of staff, insufficient resources, and the congestion of patients due to longer wait times may directly affect changes in levels of illness severity for this group^7–9^. Changes in symptoms and illness severity may lead to increased complications, morbidity, and mortality^8,10^. This is particularly true for older adults for whom an assessment is likely to show that the levels of severity can easily change from non-urgent to emergency and resuscitation^11^.

In Thailand, most hospitals have triage zones for prioritizing the patients’ level of care on arrival, including those with outpatient clinic appointments^5^. If patients have an existing clinical appointment and immediate care is not required, a nurse will perform a second assessment, either immediately or after waiting for the results of laboratory tests. Changes in severity are a known occurrence between initial triage and reassessment. Because it can be difficult for nurses to accurately assess symptoms that fluctuate, changes in severity can easily occur during the time interval. For older adults who are susceptible to RAMPS, the changes in levels of severity between the two assessment periods may cause problems with treatment planning at the clinic^1,11^.

Older adults over 65 years of age may have some alterations in their physical conditions, organ functions, and a weakened immune system^12^. One large outpatient department in northeastern Thailand reported the following numbers and percentages of patients who changed their levels of severity from non-urgency to emergency between the first and second assessment: 186 (0.48%), 261 (0.64%), and 413 (0.98%) in 2017, 2018, and 2019 fiscal years, respectively. Among those annual numbers, there were 98 (52.7%), 155 (59.4%), and 321 (77.9%) older adults^13^. During the three years, there were three non-urgent older adults whose symptoms deteriorated and required resuscitation while in the clinic’s waiting area. Although that number was low, the impact was substantial.

Other than internal hospital documents, there is little research in the published literature that explores to what extent adult and older adult patients change in severity levels and the factors related to those changes. Only the quantitative study might partially fill the gap; however, the qualitative study can answer both what and how questions. When the levels of severity change, the factors related to that change should be explored and guided for future prevention. The combination design can shed light on levels of severity change and factors related to those changes. The results from this study will guide policy change regarding the rescreening system and improve the triage system by increasing the quality of triage service for patients, especially older adults. Finally, we conducted this study to explore the factors related to severity change by applying the embedded mixed-method study.

## Aims

This study aimed to examine the incidence and changes in levels of illness severity in adult and older adult patients at an outpatient clinic. Factors related to the changes in levels of illness severity will also be explored.

## Methods

### Design

This study took an embedded mixed-method approach by first examining quantitative data collected from patients using research instruments to explore the severity level and its changes. Then, interviews with triage/assessment registered nurses (RNs) were conducted using open-ended questions in case severity changes were found to explore factors associated with the changes and provide complete reasons to answer the research questions and clarify the level of severity change phenomenon.

### Setting and sampling

The population of interest was adult and older adult patients who had scheduled clinic appointments at an outpatient internal medicine clinic from a large tertiary regional hospital in northeastern Thailand. A systematic random sample of patients (every other person) was drawn. The level of illness severity was firstly assessed at a hospital’s triage zone and secondly assessed at the internal medicine clinic by triage RNs. To ensure the triage nurse can provide the same screening quality, they should be trained, and interrater reliability should be demonstrated before conducting this study. The triage RNs had completed the Thai Ministry of Public Health’s two-day triage education training course and reached 93% agreement on severity screening (interrater reliability between 92 and 94%).

The sample size was calculated using a formula to estimate the proportion of the population with a 1.96 confidence coefficient. The proportion of patients with changes in levels of severity from a similar study was 0.476, with a tolerance of 0.05, and statistical significance set at the 0.05 level^14^. After including 10% to account for potential missing data, the estimated sample size was 421 participants, 220 adults (52.3%) and 201 older adults (47.7%). The proportion of adults to older adults was divided based on the proportion of those who received treatment at this clinic^13^.

Inclusion criteria were (a) patients aged ≥ 18 years with any health conditions, (b) triaged two times by an RN, and (c) willingness to participate in the study. The exclusion criterion was an unwillingness or inability to provide complete information.

For the qualitative data, inclusion criteria were triage nurses at the triage zone and the internal medicine clinic who gave informed consent to participate in the study. The exclusion criterion was an unwillingness or inability to provide complete information. Finally, The 18 triage nurses who found levels of severity changes were selected for interview. This criterion was set because these triage nurses could give information and details related to severity changes since they assessed the patients and had direct experience with patient severity level changes.

## Ethical considerations

The research received approval from the Khon Kaen University Ethics Committees for Human Research based on the Declaration of Helsinki and the International Conference on Harmonization (ICH) Good Clinical Practice Guidelines. The Approval number was HE642016 on March 30, 2021. Following the ethical principles of the Declaration of Helsinki, the primary investigator provided research information, and the research assistant obtained the signed informed consent forms before data collection. Finally, the data were analyzed anonymously.

### Measurements/instruments

Four research instruments were used to collect quantitative and qualitative data. An 8-item demographic data sheet collected patients’ personal and clinical data using fill-in-the-blank and a checklist. A second 8-item general questionnaire was completed by the triage RNs who assessed or reassessed the patients, also fill-in-the-blank and a checklist.

The third instrument was the Emergency Severity Index (ESI). The ESI version 4 is a triage tool to determine priorities for emergency services. The ESI consists of five levels of illness severity: 1 (*Resuscitation*), 2 (*Emergency*), 3 (*Urgent*), 4 (*Semi-urgent*), and 5 (*Nonurgent*). The interrater reliability with Cohen’s κ was 0.89^15^.

The fourth instrument was a semi-structured interview form to collect qualitative data. Two examples of open-ended questions were, “How did the severity of the patient change?” and “What were the causes of the change in severity?” All research instruments were proved by the five experts including two medical doctors, two emergency registered nurses, and one faculty member who is an expert in gerontological nursing which content validity indexes ranked between 0.90 and 1.00.

### Data collection/procedure

From April 1 to April 30, 2021, the primary investigator collected data from adult and older adult patients during their first visit to the triage zone. Then, the second screening was performed at the outpatient internal medicine clinic. If a patient had a change in the level of illness severity, the primary investigator interviewed the RN, who recorded the severity change as soon as possible to find the reason or cause of severity level changes and explain the study phenomenon. This embedded mixed-method study was used to shed light on qualitative data to deeply understand not only the incidence and changes in levels of illness severity in adult and older adult patients at an outpatient clinic but also factors or reasons related to the changes in levels of illness severity will also be explored. Finally, the average interview time was approximately 30–60 min.

### Data analysis

Quantitative data were analyzed using IBM® SPSS® version 28 under a university license. General information was analyzed using descriptive statistics, and relationships between characteristics of adults and older adults were calculated using Chi-square and Fisher’s exact tests. Three logistic regression models were performed to ascertain the effects of changes in the level of illness severity.

Qualitative data were the interviews transcribed verbatim from tape recordings. A mind-mapping software program assisted with content analysis in capturing the key points and identifying themes to understand and clarify the quantitative results.

## Results

### Demographic information

Patients' age ranged between 18 and 94 years. Most (52.0%) were female, 51.3% completed primary education, 33.6% came by ambulance, 43.2% came for clinic appointments, 51.5% had more than one chronic disease, the mean, median, and standard deviation of reassessment were 75.77, 62.00, and 37.50 min, respectively, the mean, median, and standard deviation of travel duration to the hospital were 75.00, 60.00, and 15.43 min, and 22.3% prepared unnecessary actions. Of 38 triage RNs, all were female and had bachelor's degrees, 55.3% aged 31–40 years, 95% had over ten years of service, 86.9% had never received specialized training except ESI training, 50% had experience in using the ESI tool, but about 5% had worked in the accident and emergency department (Table 1).

**Table 1:** Demographic characteristics patients and the triage nurses.

General information	Total (*n* = 421)	
	*n*	%
Age 18–94 years, (*M* = 58.90, *SD* = 17.07)		
Adults, years (range 18–59, M = 45.60, SD = 10.23)	220	52.3
Older adults, years (range 60–94, M = 73.79, SD = 8.97)	201	47.7
Gender		
Male	202	48.0
Female	219	52.0
Chronic disease		
No chronic disease	68	16.2
Have one chronic disease	136	32.3
Have more than one chronic diseases	217	51.5
Chief complaint		
Check up as appointment	182	43.2
Cardiovascular disease	92	21.9
Digestive system	37	8.8
Nervous system disorder	37	8.8
Respiratory disease	18	4.3
Infectious disease	16	3.8
Kidney disease	12	2.9
Cancer	12	2.9
Arthritis	8	1.9
Skin disease	4	0.95
Blood and hematological disorders	2	0.5
Endocrine disease	1	0.2
Educational level		
Uneducated	29	6.9
Primary school	186	44.4
Secondary school	93	22.0
Vocational certificate	24	5.7
High vocational certificate/Diploma	23	5.4
Bachelor’s degree	58	13.7
Master’s degree/Ph.D	8	1.9
Reassessment interval (M = 75.77, Median = 62.00, SD = 37.50 min)		
0–60 min	199	47.3
Longer than 60 min	222	52.7
Duration of travel to the hospital (M = 75.00, Median = 60.00, SD = 15.43 min)		
1–60 min	250	59.4
61–120 min	118	28.0
121–180 min	53	12.6
Self-preparation before coming to the hospital		
Nothing by mouth (N.P.O.), without recommendation	25	5.9
Prepare for special examination	6	1.4
Take personal medicines	321	76.3
Did not take personal medicines, without recommendation	69	16.4
Type of vehicle used to come to the hospital		
Ambulance referral service	140	33.6
Private car	128	30.2
Bus	80	18.9
Motorcycle	73	17.3
Gender		
Female	38	100
Age years: range 31–57, *M* = 39.1, *SD* = 6.01		
31–40 years	21	55.3
41–50 years	10	26.3
51–60 years	7	18.4
Nursing positions		
Registered nurse (practitioner level)^a^	3	7.9
Registered nurse (professional level)^a^	35	92.1
Education		
Bachelor’s degree	38	100
Specialized training		
None	33	86.9
General nurse practitioner training	2	5.3
Triage training	1	2.6
More than one training course (ACLS, triage)	1	2.6
Training in gerontological nursing	1	2.6
Years of service		
1–5 years	0	0
6–10 years	2	5.2
11–15 years	9	23.7
16–20 years	9	23.7
21–25 years	5	13.2
25–30 years	7	18.4
31–35 years	4	10.5
36–40 years	2	5.3
Experience in accident & emergency department		
None	36	94.7
1–5 years	1	2.6
6–10 years	1	2.6
Experience in using the Emergency Severity Index		
1–5 years	19	50.0
6–10 years	19	50.0

^a^Practitioner and professional registered nurses are the first two steps in a hospital clinical ladder for promotion and benefits.

M = Mean; S.D. = Standard Deviation; Ph.D. = Doctor of Philosophy; ACLS = Advanced Cardiovascular Life Support; *n* = Number.

### Patients’ triage assessments

The most common ESI levels for both adult and older adult patients for the first and second times were level 4 (n = 270 & n = 214, Nonurgent), followed by level 3 (n = 146 & n = 176, Semi-urgent), respectively (Table 2). The level of severity changes were divided into two subgroups: increased and decreased. Among the older adults, 89 (44.3%) increased their level of severity (Table 3).

**Table 2:** Emergency severity index classification of patients at the first and second assessments.

Screening	ESI level 2		ESI level 3		ESI level 4		ESI level 5	
	*n*	%	*n*	%	*n*	%	*n*	%
1st assessment	–	–	146	34.7	270	64.1	5	1.2
2nd assessment	26	6.2	176	41.8	214	50.8	5	1.2

ESI = Emergency Severity Index; *n* = Number.

**Table 3:** Changes in the levels of severity between the first and the second assessments.

Patient age categories	*n*	%	Unchanged symptoms		Changes in levels of severity			
					Increased severity		Decreased severity	
			*n*	%	*n*	%	*n*	%
Combined	421		260	61.8	114	27.1	47	11.2
Older adults	201	47.7	86	42.8	89	44.3	26	12.9
Adults	220	52.3	174	79.1	25	11.4	21	9.6

*n* = Number.

### Quantitative results

#### Factors related to changes in levels of severity

Older adults were over five times more likely to experience changes in severity than adult patients. Patients having more than one chronic disease, coming to the hospital when experiencing symptoms related to diseases, spending a longer travel time, reassessment interval over an hour, arriving by ambulance, and arriving after 12:01 for the triage, were more likely to have changes in levels of illness severity. Factors related to increased severity were also the same. Moreover, patients who were less educated were four times more likely to have increased levels of severity. Finally, factors related to decreased severity were older adults and having more than one chronic disease (Table 4).

**Table 4:** Changes in patients’ levels of severity, increases or decreases in severity, and factors related to the changes.

Factor	Changes in levels of severity (n = 421)		Chi-Square or Fisher’s Exact test			Increased levels of severity (n = 374)		Chi-square or Fisher’s Exact test			Decreased levels of severity (n = 307)		Chi-Square or Fisher’s Exact test		
	Unchanged severity (n = 260) n (%)	Changed severity (n = 161) n (%)	${x}^{2}$	OR (95%CI)	*p*	Unchanged severity (n = 260) n (%)	Changed severity (n = 161) n (%)	${x}^{2}$	OR (95%CI)	*p*	Unchanged severity (n = 260) n (%)	Changed severity (n = 161) n (%)	${x}^{2}$	OR (95%CI)	*p*
Type			58.62		0.01			64.44		0.01			8.50		0.01
Older adult	86 (42.8)	115 (57.2)		5.05 (3.22–7.95)		86 (49.1)	89 (50.9)		7.2 (4.2–12.5)		86 (76.8)	26 (23.2)		2.5 (1.3–5.0)	
Adult	174 (79.1)	46 (20.9)		1		174 (87.4)	25 (12.6)		1		174 (89.2)	21 (10.8)		1	
Gender			0.02		.89			.32		.57			.42		.52
Male	124 (61.4)	78 (38.6)		1.0 (.7–1.6)		124 (68.1)	58 (31.9)		1.13 (0.7–1.8)		124 (86.1)	20 (13.9)		1.2 (.6–2.4)	
Female	136 (62.1)	83 (37.9)		1		136 (70.8)	56 (29.2)		1		136 (83.4)	27 (16.6)		1	
Chronic disease			240.06		0.01			21.41		0.01			7.43		0.02
None	53(77.9)	15(22.1)		1		53 (85.5)	9 (14.5)		1		53 (89.8)	6 (10.2)		1	
1 disease	97(66.9)	39(33.1)		4.8 (2.3–10.1)		97 (77.6)	28 (22.4)		1.7 (.7–4.4)		97 (89.8)	11 (10.2)		1.00 (.3–3.5)	
> 1 disease	110(50.7)	107(49.3)		14.8 (7.6–29.8)		110 (58.8)	77 (41.2)		4.1 (1.9–10.0)		110 (78.6)	30 (21.4)		2.4 (.9–7.5)	
Chief complaint			9.97		0.01			15.75		0.01			0.01		.97
Appointment	128(70.3)	54(29.7)		1.9 (1.3–3.0)		128 (80.5)	31 (19.5)		2.6 (1.6–3.3)		128 (84.8)	23 (15.2)		1.0 (.5–2.0)	
All diseases	132(55.2)	107(44.8)		1		132 (61.4)	83 (38.6)		1		132 (84.6)	24 (15.4)		1	
Education			2.86		0.09			8.41		0.01			1.05		.31
< Bachelor’s degree	225(60.3)	148(39.7)		1.8 (0.9–3.8)		225 (67.2)	110 (32.8)		4.3 (1.5–16.9)		225 (85.5)	38 (14.5)		.7 (.3–1.7)	
≥ Bachelor’s degree	35(72.9)	13(27.1)		1		35 (89.7)	4 (10.3)		1		35 (79.5)	9 (20.5)		1	
Travel time			16.26		0.01			15.75		0.01			4.33		.12
0–60 min	173(69.2)	77(30.8)		1		173 (76.5)	53 (23.5)		1		173 (87.8)	24 (12.2)		1	
61–120	64(54.2)	54(45.8)		1.9 (1.2–3.0)		64 (62.7)	38 (37.3)		1.9 (1.1–3.3)		64 (80.0)	16 (20.0)		1.8 (.8–3.8)	
121–180	23(43.4)	30(56.6)		2.9 (1.5–5.6)		23 (50.0)	23 (50.0)		3.3 (1.6–6.6)		23 (76.7)	7 (23.3)		2.2 (.7–6.0)	
Preparation			3.77		0.05			5.460		.20			.39		.84
Incorrect	70(70.0)	30(30.0)		1		70 (79.5)	18 (20.5)		1		70 (85.4)	12 (14.6)		1	
Correct	190(59.2)	131(40.8)		1.6 (1.0–2.7)		190 (66.4)	96 (33.6)		2.0 (1.1–3.7)		190 (84.4)	35 (15.6)		1.8 (.5–2.4)	
Travel by			20.06		0.01			29.61		0.01			.11		.74
On their own	194(69.3)	86(30.7)		1		194 (78.9)	52 (21.1)		1		194 (85.1)	34 (14.9)		1	
Ambulance	66(46.8)	75(38.2)		2.61.7–4.0)		66 (51.6)	62 (48.4)		3.5 (2.1–5.7)		66 (83.5)	13 (16.5)		1.1 (0.5–2.3)	
Triage			10.32		0.04			23.50		0.01			6.04		.20
6:00–8:00	88(67.7)	42(32.3)		1		88 (75.2)	29 (24.8)		1		88 (87.1)	13 (12.9)		1	
8:01–10:00	120(64.9)	65(35.1)		1.1 (.7–1.9)		120 (77.4)	35 (22.6)		0.9 (.5–1.6)		120 (80.0)	30 (20.0)		1.7 (.8–3.7)	
10:01–12:00	10(55.6)	8(44.4)		1.7 (.5–5.1)		10 (58.8)	7 (41.2)		2.1 (.6–6.8)		10 (90.9)	1 (9.1)		.7 (0–5.5)	
12:01–13:00	33(47.8)	36(52.2)		2.3 (1.2–4.3)		33 (49.2)	34 (50.8)		3.1 (1.6–6.2)		33 (94.3)	2 (5.7)		.4 (0–2.0)	
13:01–14:00	9(47.4)	10(52.6)		2.3 (.8–7.0)		9 (50.0)	9 (50.0)		3.0 (1.0–9.5)		9 (90.0)	1 (10.0)		.8 (0–6.3)	
Reassessment		7.28		.12			19.40		0.01			7.84		.10	
6:00–8:00	8(53.3)	7(46.7)		1.7 (.5–5.7)		8 (61.5)	5 (38.5)		2.3 (.6–8.4)		8 (80.0)	2 (20.0)		1.1 (.1–5.8)	
8:01–10:00	143(66.5)	72(33.5)		1		143 (78.6)	39 (21.4)		1		143 (81.2)	33 (18.8)		1	
10:01–12:00	30(60.0)	20(40.0)		1.3 (.7–2.6)		30 (69.8)	13 (30.2)		1.6 (.7–3.5)		30 (81.1)	7 (18.9)		1.0 (.3–2.6)	
12:01–13:00	44 (62.9)	26 (37.1)		1.2 (.6–2.1)		44 (65.7)	23 (34.3)		1.9 (1.0–3.7)		44 (93.6)	3 (6.4)		.3 (.1–1.0)	
13:01–14:00	35 (49.3)	36 (50.7)		2.0 (1.1–3.7)		35 (50.7)	34 (49.3)		3.6 (1.9–6.7)		35 (92.1)	2 (7.9)		.4 (.1–1.3)	
Reassessment interval		16.35		< .001			21.63		< .001			2.46		.12	
0–60 min	98 (49.3)	101 (50.7)		2.45 (1.6–3.7)		74 (42.3)	101 (57.7)		2.91 (1.8–4.6)		24 (19.2)	101 (80.8)		1.6 (.9–3.1)	
Longer than 60 min	63 (28.4)	159 (71.6)				40 (20.1)	159 (79.9)				23 (12.6)	159 (87.4)			

OR = Odd Ratio; CI = Confident Interval; χ^2^ = Chi-square; n = Number; *p* = *p*-value.

#### Factors predicting levels of illness severity changes

The first logistic regression model was statistically significant and explained 23.2% (Nagelkerke *R*^2^) of the variance in the level of severity change and correctly classified 73.2% of cases. Factors with significant odds ratios affecting change in the level of illness severity were age (older adults), having more than one chronic disease, coming to the hospital when experiencing symptoms related to diseases, reassessment interval over an hour, and time of the reassessment later in the day/afternoon. A second logistic regression model examined factors associated with an increase in severity levels. The model was statistically significant and explained 33.3% (Nagelkerke *R*^2^) of the variance and correctly classified 76.2% of cases. Factors affecting an increase in severity level were being an older adult, coming to the hospital when experiencing symptoms related to diseases, reassessment interval over an hour, and the time of the reassessment. Unfortunately, the third logistic regression model examined neither being an older adult nor having a chronic disease was statistically significant (Table 5).

**Table 5:** Factors predicting any change in the levels of illness severity, and increased or decreased levels of illness severity.

Factors	*n*	Odds ratio	95% Confidence interval	*p*
Model 1: Factors predicting changes in levels of illness severity^†^				
Adults (18–59 years) or older adults (≥ 60 years)	421	5.05	3.22, 7.95	< 0.001
Chronic disease (none, one, or > one)	421	4.8	2.3, 10.1	0.045
Chief complain (appointment or all disease)	421	1.9	1.3, 3.0	0.014
Travel time (0–60, 61–120, 121–180 min)	421	2.9	1.5, 5.6	0.115
Type of vehicle (ambulance or other)	421	2.6	1.7, 4.0	0648
Time of reassessment (6:00–8:00 a.m., 8:01–10:00 a.m., 10:01–12.00 a.m., and 12:01–14:00 p.m.)	421	2.3	0.8–7.0	0.012
Reassessment interval (0–60 min, > 60 min)	421	2.45	1.6–3.7	< 0.001
Model 2: Factors predicting increased levels of illness severity^†^				
Adults (18–59 years) or older adults (≥ 60 years)	374	7.2	4.2, 12.5	< 0.001
Chronic disease (none, one, or > one)	374	1.7	0.7, 4.4	0.284
Chief complain (appointment or all disease)	374	2.6	1.6, 3.3	0.027
Patients’ education (< bachelor’s degree or ≥ Bachelor’s degree)	374	4.3	1.5, 16.9	0.552
Travel time (0–60, 61–120, 121–180 min)	374	3.3	1.3, 6.6	0.130
Type of vehicle (ambulance or other)	374	3.5	2.1, 5.7	0.215
Time of reassessment (6:00–8:00 a.m., 8:01–10:00 a.m., 10:01–12.00 a.m., and 12:01–14:00 p.m.)	374	3.0	1.0, 9.5	< 0.001
Reassessment interval (0–60 min, > 60 min)	421	2.91	1.8–4.6	< 0.001
Model 3: Factors predicting decreased levels of illness severity^†^				
Adults (18–59 years) or older adults (≥ 60 years)	307	2.5	1.3, 5.0	0.266
Chronic disease (none, one, or > one)	307	1.0	0.3, 3.5	0.220

^†^Model 1: χ^2^_(8, *n* = 421)_ = 73.20, *p* < 0.001; Model 2: χ^2^_(9, *n* = 374)_ = 76.20, *p* < 0.001; Model 3: χ^2^_(3, *n* = 307)_ = 8.68, *p* = 0.03.

n = Number*; p* = *p* value.

#### Qualitative findings: factors related to changes in levels of severity

Based on the quantitative results, the causes of levels of severity change were deeply explored to explain the phenomenon. Findings from quantitative results and the in-depth interview were merged into the themes to explain why they were changed in severity levels. These themes included the aging process and comorbidity, unstable illness, reassessment interval, nurses’ clinical experiences, bypassing the initial triage process, self-preparation of patients, management of triage RNs, and assigned nurse staff. The reasons and coding words for each theme were provided as follows.

#### Aging process and comorbidity

The quantitative results indicated that changes in severity levels, significantly increased levels, were found among older patients. In-depth interviews explored this change, and it found that atypical presentations associated with the five clinical features (RAMPS) were not detected in some older adults at the hospital’s triage zone and caused difficulties and inaccuracies in the RNs’ deciding the appropriate severity level. Changes were found because patients had a reduced energy reserve, multiple chronic diseases, and unclear symptoms at the time of admission.

This elderly patient with comorbidity did not show any obvious severe symptoms at first, but when waiting for the blood results, it was found that the symptoms were not the same--increased fatigue, drowsiness, and a thready pulse. We needed to send the patient to the emergency room. The risk is likely to come from the vulnerability of aging and comorbidity. Changes in symptoms can occur easily in older adults. (NU03056413)

#### Unstable illness

Changes in the levels of illness severity may have been caused by the unstable condition of the illness’ pathology, especially cardiovascular and respiratory diseases. The instability was likely to have increased the severity between the two assessment periods.

When I assessed at the time of admission, the patient did not show any abnormalities. During the waiting period, the patient's symptoms changed-sweating and drowsiness. The relatives told that the patient did not stop taking medicines, and symptoms were normal. Therefore, I think [the changes were] due to [the] severe pathology of the disease. The most common cases were patients with heart and lung diseases. The time of the increased severity is quite unpredictable. (NU05056405)

#### Reassessment interval

Changes in severity level, especially increased level of severity, were found in the duration between the first and the second time over an hour. Assessment of levels of severity should be periodically monitored because changes in severity can occur at any time, especially during waiting time. Reassessment interval should be minimally delayed for patient safety. The in-depth interview found that this interval affected changes in severity.

Reassessment is critical in the screening process and treatment planning. There were occasions when resources were insufficient to serve recipients under normal operations. The other day, some staff members were on leave, but there were a lot of [patients]. It took almost an hour to call the patients for reassessment. [Because of the] delay, the severity of the patient’s disease had progressed-possibly resulted in the increased level of severity. If the symptoms could be reassessed early, changes would be detected earlier. (NU05056405)

#### Nurses' clinical experiences

Nurses' experiences and understanding of both the ESI tool and older adults’ clinical features could have resulted in a more accurate assessment and improved the ability to anticipate and manage care appropriately. This could have reduced the changes in the severity of patients.

The other thing is the experience of predicting the symptoms. The anticipation for patients with cardiac symptoms was that it was riskier for patients to walk to each service point. Patients should be provided with a wheelchair or a stretcher because they required several steps of treatment. The outpatient building is quite large, so walking to each point can make patients feel tired. Patients should stay in a wheelchair or a stretcher to prevent changes in symptoms [and] to enhance safety. [Patients who] receive care like this usually have no changes in their levels of severity. (NU04056404)

#### Bypassing the initial triage process

Some patients bypassed the triage zone and went directly to the internal medicine clinic. In doing so, they missed the assessment of illness severity so that appropriate care and referral services could be given. However, on arrival at the clinic, they received an initial assessment that was re-evaluated while waiting in the queue. Because of delays in waiting, levels of severity could have increased.

Patients were bypassing the first screening point. The nursing staff was unable to thoroughly provide care, causing patients and their relatives not to realize that their symptoms were at a level of urgency. In addition, the symptoms became worse before the staff had made a reassessment. (NU07056413)

#### Self-preparation of patients

Preparing for the clinic exam before traveling to the hospital was a factor affecting an increased severity. Although there was no statistical difference in the quantitative results, there was a noticeable clinical effect. Over 40% of the patients who improperly prepared for their clinic visit experienced worsening symptoms.

The patient was sent from a community hospital for an endoscopy and abstained from water and food on the expectation that the doctor would perform an endoscopy that day. However, it was necessary to diagnose first, not to do it right away. [On arrival at] the hospital, the patient fainted because of hypoglycemia (Dtx=45 mg%). Another case was the patient forgot to take the medications for high blood pressure because he had to wake up at 4:00 a.m. to queue early for avoiding not having a car to go home. His BP was 190/110 mmHg. Both needed emergency care (NU11056410)

#### Management of triage RNs

At the triage zone, the key issue was assessing for life-threatening conditions. When patients were found to be at risk of a change in severity, the management by triage RNs was placing triage tags on the patients. They would be monitored during their time and this management caused a positive impact on patient safety.

One patient with late-stage lymphoma was bedridden with a tracheostomy. His ESI was almost level 2 because of the risk of hypoxia. The patient was placed with a triage tag, and the information was forwarded to the first aid room. Oxygen was given and symptoms had been monitored. For this management, there was no increase in severity. Communicating information at each point is of great importance to prevent changes in severity. (NU27056415)

#### Assigned nursing staff

In some patients, the level of severity was borderline at the triage zone, i.e., symptoms did not meet all criteria needed to be sent to the emergency room. For these individuals, it was necessary to assign someone to watch them carefully and speed up the process from admission through the end of treatment. This could ensure patient safety and the result would be a positive outcome.

This patient was triaged at ESI almost level 2. The symptoms had not yet been stabilized, but he had to wait to see a cardiologist. The staff had already coordinated with one another. The method of assigning staff to take care of him and enter the fast track was used to reduce the waiting time and prevent changes. Based on the reassessment, the severity level had not changed. He had received proper care [throughout] the treatment. The symptoms had been relieved without increased severity. (NU17056417)

## Discussion

We found that most patients with increased severity were older adults. This is consistent with other studies that have reported similar findings^16,17^. Our qualitative results also show that changes in symptoms can occur easily in older adults. Older patients with comorbidities did not show any obvious severe symptoms at first, but the symptoms were not the same during the waiting time. The change is likely to come from the vulnerability of aging and comorbidity. Because of the aging process, older people tend to have changes in increased severity due to having a reduced energy reserve and fragility^1^. Suamchaiyaphum et al. ^10^ reported that age influences severity assessment and that age could predict the possibility of different outcomes at reassessment. In another study, up to 34% of the participants aged 65–84 changed their level of severity at reassessment by 1.71 times, compared to other age groups^17^.

Most of the older adults in our study had multiple pathologies with one or more chronic diseases. Patients with chronic diseases or comorbidities were more likely to increase their severity level at reassessment. This is consistent with Hasadsree et al. ^18^ who found that comorbidities resulted in greater severity and shock. Similarly, Suamchaiyaphum et al. ^10^ reported that two or more comorbidities had an increased impact on illness severity and pathology. Mirhaghi et al. ^19^ also found that comorbidities and various symptoms affected the patient triage, changes in symptoms, and treatment difficulties.

Symptoms related to cardiovascular and respiratory diseases as chief complaints were important factors affecting changes in severity. The quantitative results found that patients with heart and lung diseases were the most common cases with changing severity levels. The time of the increased severity is quite unpredictable for these health problems. Hinson et al. ^17^ found that the chief complaints of syncope, chest pain, and dyspnea were associated with changes in severity. Krongthong et al. ^20^ and Suamchaiyaphum et al. ^10^ confirmed that the chief complaint related to the cardiovascular and respiratory systems correlated with changes in severity. Therefore, if the chief complaint is related to impaired cardiovascular and respiratory systems, there should be a fast track for this group of patients to reduce worsening severity levels.

The patient's educational level was related to changes in levels of severity. People with low levels of education often have obstacles in accessing medical services. These include poor reading and listening skills, failure to listen to advice on treatment protocols and fear of being scolded by service providers. This can result in either miscommunication or the perception of misinformation that leads to unproductive and unhealthy behaviors^21^. Nilnate et al. ^22^ reported that health literacy scores among older adults were low to moderate. They had deterioration of their ears and eyes that can affect self-care management skills and competency for accessing, understanding, and reporting health knowledge. These circumstances might affect the process of history taking during triage, causing incomplete health data and determination of the correct ESI level.

We found that patients who came to the internal medicine clinic with a long journey and had long intervals between triage and reassessments resulted in changes in illness severity. Time length in traveling plus a long wait period may cause fatigue, a patient deterioration, and require earlier service procedures. This is consistent with the study of Hasadsree et al. ^18^ who found that the duration of travel/being transferred to the hospital that took longer than 60 min resulted in changes in illness severity and were related to shock. Another study also found that a prolonged time interval between assessments results in delays and adverse effects on treatment, leading to changes in severity^23^. The reassessment should be completed within 60 min of the first assessment, and ongoing observation and monitoring of symptoms would have a positive effect on detecting changes in severity.

We found that patients who arrived by referral ambulance were more vulnerable to changes in their symptoms. Similarly, Suksawang ^24^ reported that patients who came by emergency medical services or referral ambulance had the severity of symptoms at ESI levels 1 and 2. These patients required medical attention and close monitoring from RNs.

The clustering of patients at certain appointment times limited the triage RNs’ ability to assess them effectively. Arkun et al. ^25^ also found that there was only one triage RN at the facility at certain times because the RNs needed to leave for lunch or take a break. This finding is similar to our qualitative result in that some staff members were on leave, but there were many patients. It took almost an hour to call the patients for reassessment. Having many patients and a limited number of nurses produced delays in patient triage and a decrease in the effectiveness of patient triage, causing changes in illness severity.

The qualitative findings showed that nurses' clinical experiences affected decision-making and accurate prediction of the symptoms. In Thailand, new nurses with no experience are not assigned to triage the patients; they should have experience in nursing and be prepared to be sensitive to serious health problems and perform accurate screening results to detect and help patients with acute and emergency health problems. This finding is consistent with the study of Tonsaur et al. ^26^ and Wachiradilok et al. ^9^, who reported that staff members who had less than five years of service never used the ESI tool nor had been in a unit using the ESI tool for patient triage, might lack confidence in performing accuracy of triage. Those with more experience working and using the ESI tool could use it proficiently, make accurate assessments, anticipate what might happen to the patients, and create a management plan more efficiently.

Some patients who bypassed the triage zone reported changes in their levels of illness severity. Our qualitative results found that because patients were bypassing the first screening point, the nursing staff was unable to thoroughly provide care, causing patients and their relatives not to realize that their symptoms were at a level of urgency. In addition, the symptoms became worse before the staff had made a reassessment. Thesprasit ^27^ also reported that patients who were not initially triaged had changes in severity and developed worse symptoms while waiting for examination. Patients who provided inappropriate self-preparation before arriving at the clinic experienced changes in severity levels. Some patients from this study abstained from water and food before being admitted to the hospital for endoscopy. However, it was necessary to diagnose first, not to do it on the first day of admission. The abstaining from water and food caused their severity to increase during the waiting time. Suksawang ^24^ also reported that more than half of the patients in a study had improper practices before coming to the hospital, so their symptoms worsened while waiting for treatment.

Good management and the assignment of nursing staff to observe specific patients until the end of the treatment process can have a positive effect on safety and may reduce the severity of the patient’s symptoms. Although the triage process has clear operating guidelines, Suksawang ^24^ identified desirable characteristics of triage RNs. When immediate problems arise, triage RNs should remain flexible and manage solutions that focus on the benefit and safety of the patients. Good management by RNs for patients with late-stage lymphoma who are bedridden with a tracheostomy was demonstrated by our study. Good management promptly provided safety for the patients.

## Limitations

Because the ESI tool guidelines do not specify the interval between triage and reassessment time nor the appropriate time for reassessment at each severity level, we conducted the study in an outpatient department context by reassessing immediately upon the patient's arrival at the internal medicine clinic. Therefore, the interval between the triage of the patient and reassessment varied for each patient.

## Conclusion and recommendations

**Figure 1:**
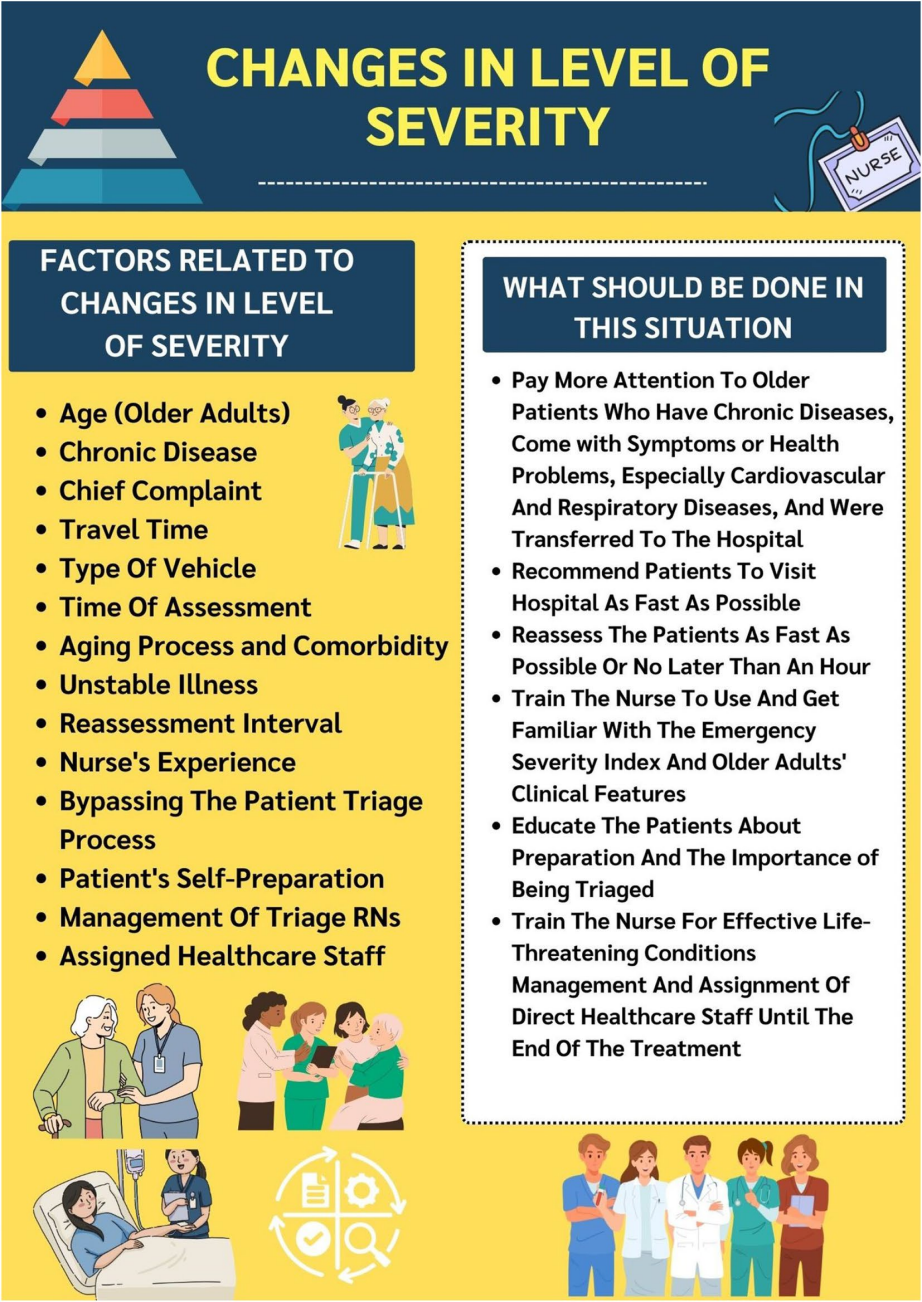
Demonstrate factors related to changes in level of severity and what should be done in this situation.

Although most of the patients had no change in severity, those with changes that increased their level of severity were more frequently found in older adults. Factors related to changes in severity from both quantitative and qualitative data were age group, chronic disease, chief complaint, travel time, type of vehicle, time of assessment, aging process and comorbidity, unstable illness, reassessment interval, nurse's experience, bypassing the patient triage process, patient's self-preparation, management of triage RNs, and assignment of direct healthcare staff until the end of the treatment. The factors related to changes in level of severity and what should be done in this situation were summarized and provided (Fig. 1).

This study made reassessment immediately when patients arrived at the internal medicine clinic. Future studies should control or determine a time for reassessment clearly and consistently. Alternatively, different times of reassessment may be explored as an important variable for the outcome analysis, especially in older adults. In addition, the results of the study could form the basis of a nursing staff education plan to increase knowledge, competence, and awareness of aging processes about the assessment of severity levels. The ESI tool may need to be refined for more sensitivity in assessing older adults.

## Data Availability

The datasets generated and/or analyzed during the current study are not publicly available due to prohibited laws (and/or rules, regulations, and contracts). However, they are available from the corresponding author upon reasonable request.
